# Miscellanies

**Published:** 1844-07-01

**Authors:** 


### Miscellanies.

### The Naval Lunatics at Haslar.

In no situation has the humane system of treating lunatics been carried out with greater vigour than in the Royal Naval Hospital at Haslar, and nowhere with more satisfactory and gratifying results. So soon as the happy consequences of Dr. Conolly's new system at Hanwell came to the knowledge of the Director-General of the Medical Department of the Navy, Sir William Burnett, he determined on introducing it into the Government Asylum at Haslar; and he had the good fortune to find in one of the officers of his own service, Dr. Anderson, a man not merely fully convinced of its immense superiority, but practically conversant with its details. For some time previously to his appointment, Dr. Anderson had been the resident physician of the well-known private institution at Denham Park, near Uxbridge, where the non-restraint system was and is carried to its full extent. At the period of Dr. Anderson's appointment, the naval lunatics, both men and officers, were treated pretty much in the old plan, although Sir William Burnett had made strenuous efforts for the introduction of the more rational system. Dr. Anderson's arrival at Haslar was the epoch of a complete reform; and as his views coincided in every respect with those of Sir William Burnett, and as that officer had the entire confidence of the Lords of the Admiralty (who entered into the question with a degree of zeal and liberality highly honourable to themselves), every projected improvement was carried into effect with true nautical energy. The change for the better was the more remarkable for its rapidity. Those who had known the asylum under the old rule could scarcely credit their senses when witnessing, after a few months, the new discipline of the place and the new habits and dispositions of the inmates. Chains, straps, corsets, imprisonment—all vanished at the will of the Superintendent;—and the false fears of the attendants, and much of the gloom and misery of the patients, soon followed. This altered condition of things has now existed about two years, and not a single accident has occurred to checker the satisfaction of those who brought it about.

Among the happy changes introduced by Dr. Anderson was one suggested by Sir William Burnett, which does great credit to his humanity, and has been a source of immense gratification to the patients. The airing-court attached to the asylum being at the back of the hospital, which stands on flat ground, was necessarily deprived of all prospect of the surrounding country, and even of the neighbouring sea. To remedy this defect it was determined to erect a lofty mound in the centre of the airing-ground; and this was no sooner suggested than it was carried into effect by the lunatics themselves, under the direction of their benevolent superintendent. This mound is of large dimensions, solidly and beautifully constructed, and furnished with a gravel-walk leading by a gentle slope to the summit. It is sufficiently lofty to command a very extensive and beautiful view of the Isle of Wight as far as Cowes and St. Helen's, the towns of Portsmouth and Portsea, great part of Portsmouth harbour, all Spithead, and the neighbouring sea, with the ever-busy and ever shifting panorama of masts and sails and flags that crowd that nautical thoroughfare. It is impossible to contemplate, without emotion, the happy influence that such a change as this must have had on the feelings of the sailors. The famous shout of Xenophon's soldiers, "The Sea! the sea!" seems again realized to the imagination when we think of the feelings that may have stirred the withered souls of those solitary men, when, after years of imprisonment within gloomy walls, they were again, as it were, restored to their old element. How much they prize the privilege thus accorded by the purest and most refined humanity, is proved by the continued eagerness displayed by them in climbing this "sacred mount." A similar elevation is now being built in the grounds of the officers' department.

A still bolder step in the progress of the rational and humane treatment of these poor fellows, and, we doubt not, in the cure of their bruised and broken minds,—an improvement which strikes us almost as much by its happy boldness as by its genuine benevolence,—has been more recently introduced. A boat has been granted to them by the Admiralty; and in this they may now be seen pulling and steering their fearless and noble-hearted friend and master, Dr. Anderson, not only through Portsmouth harbour, but actually out to sea, calmly enjoying the cooling breeze, or busy in their long-forgotten pastime of fishing.

But the account of this affecting experiment, and a few more interesting particulars of the proceedings of the Naval Asylum, we are enabled, through the kindness of Sir William Burnett, to give in Dr. Anderson's own words: and we cannot present them to our readers without offering to that gentleman the tribute of our respect and gratitude for his enlightened and noble exertions.

*Extract from Dr. Anderson's Report, Midsummer Quarter,* 1843.—"The calm and orderly conduct which now prevails throughout the asylum, together with the cleanly, and I may add industrious, habits of a large proportion of the patients, render the duties of the attendants and nurses comparatively easy. For the accomplishment of these desirable ends I have endeavoured to carry out the excellent precepts laid down by Dr. Conolly of Hanwell, and in order to point out clearly the principles by which the medical officers themselves are guided, and that they constantly inculcate on the attendants and nurses, I cannot do better than quote the following paragraph from one of that talented physician's Reports. 'To endeavour to gain and preserve the confidence of each patient; to create or maintain a character of kindness and tranquillity throughout the asylum; to forbid the exercise of violence, threats, or deception; to be careful of their diet and clothing; to occupy and amuse them; to secure their cheerfulness or content by day and comfortable rest at night; to consider all their weakness and infirmities; and to pay a general regard to whatever may act favorably on the mind and body.' These have been and continue to be the principles on which our moral management is based, and the fruits of this mild and rational treatment are now clearly developed in the altered condition of our inmates, whose conduct for many months past has very generally been characterized by a constant and orderly demeanor, which I am persuaded no coercive measures could ever have produced. The means of recreation and exercise which have wisely been extended to the patients in allowing them to walk into the surrounding country, continue to be a source of great delight to many; and nothing is felt as a more severe punishment than this salutary freedom being withheld from any of those who are usually in the habit of joining the party in these country excursions. The cheerful aspect of the new airing-grounds with the mound in the centre, has been very much increased by the late alterations; and being now thrown open in fine weather, have become the daily resort of a large proportion of the patients. The inducement thus offered to the indolent and melancholic to take a view of Spithead, Portsmouth harbour, Isle of Wight, and the surrounding country, is found not only to form the means of amusement and exercise, but, it may almost be said, some alleviation of their malady.

"The religious services continue to be regularly performed in the asylum morning and evening; and it is rather surprising to find between sixty and seventy insane persons assembled together for the purpose of divine worship without the occurrence of any disturbance, except on very rare occasions. Upwards of twenty attend the service in the chapel every Sunday; and their conduct during divine worship has been uniformly marked by the same decorum as the sane part of the congregation.

"In consequence of directions from the Inspector-General, the dress for the seamen and marines was changed at Easter, the whole of the lunatic patients being now supplied with blue clothing instead of the brown formerly worn. This change to their favorite colour has given the seamen great satisfaction; and no doubt, has in some of the number, brought back to their recollection early and pleasing associations.

"The many alterations, sanctioned and promoted by the Inspector-General, which have taken place generally throughout the asylum during the last twelve months, have contributed in no small degree to the improved condition of the patients; and when the works now in progress are completed, the whole face of the establishment will be changed from its former gloomy and prison-like appearance to a cheerful place of residence, which will neither be calculated to create alarm in the timid and suspicious, nor excite the rage of the more furious madman."

*Extract from Dr. Anderson's Report; Michaelmas Quarter*, 1843.—"The Lords of the Admiralty have very recently, on the recommendation of Sir William Burnett, furnished a boat for the use of the lunatic patients; and since it has been received from the dockyard they have had many rowing and several sailing excursions, both in the harbour and out to Spithead. Upwards of twenty of the patients have, at different times, joined in this amusement; and on every occasion they have behaved in the most quiet and orderly manner, and can now, after the practice of some weeks, manage the boat either under sail or in rowing with perfect ease and dexterity. I shall not attempt to describe the enjoyment which many have felt by being again permitted to embark on their favourite element, nor the pleasure with which they look forward to these water-excursions. Some of those who have proved to be our best boatmen have been confined within the narrow limits of the asylum airing-grounds for upwards of twenty years; but notwithstanding this protracted period of seclusion, their new occupation appears to have roused mental energies which to the common observer were nearly extinct, and to have brought back to their enfeebled minds old and pleasing associations which have been productive of the most beneficial results. I have no hesitation in stating, that of all the remedial agents with which we have during the last eighteen months been so liberally supplied, the use of the boat for many of our patients is, beyond all comparison, the most valuable of any; and my anticipations, sanguine as they were, as to the benefits likely to result from that kind of exercise and recreation on the mind of the lunatic sailor, have been most fully and completely realized.

"The Inspector-General has also directed a supply of fishing lines and hooks to be furnished, and the patients have frequently, during the last month, been successfully occupied in fishing, which is also a source of great enjoyment and interest to many. Mr. * * * affords, among many others, a striking example of the salutary influence resulting from the new occupation. This officer has been in the asylum for eight years, and during the last six has very rarely spoken to any one, and appeared to take little or no notice of surrounding objects. The various means that were had recourse to with the view of rousing him from his apathetic state proved fruitless. He, in common with others, was furnished with a fishing line; and on his first trip to the buoy of the Boyne, which is our principal fishing ground, caught nine whiting-pout, and enjoyed the sport as intensely as any one in the boat, baiting his hooks himself, and making observations on his success. On our way home he took all the fish that were caught out of the basket and *counted them aloud,* much to the astonishment of Mr. Steward and myself, as well as to others who had scarcely heard the sound of his voice for several years. It is a pleasing part of my duty to be thus enabled to report the complete success of a measure which, in the estimation of many, was fraught with so much personal danger to the lunatic, by affording him an easy opportunity of carrying any suicidal propensity into effect; but the truth is, that in well-regulated establishments for the reception of the insane, the tendency to self-destruction is nearly, if not altogether, as rare as amongst those who are considered the sane part of the community.

"It is scarcely necessary to state that no bodily restraint has been had recourse to; but, as was explained to the Inspector-General during his recent official visit, it has frequently happened that for many days in succession not a single individual has been placed in temporary seclusion, thus clearly demonstrating the many advantages resulting from a mild and conciliatory system of management as compared with harsh and coercive measures.

"In conclusion, I may be permitted to state the extreme gratification I have felt in witnessing the progressive but steady improvement in the general demeanor, the cleanly habits, and the contented appearance of many whose condition, twelve months ago, scarcely held out, under any treatment, the prospect of such cheering results."

We think that no one, after reading the foregoing account, will hesitate to place the name of ANDERSON high on the list of those noblest philanthropists who are proud to own Dr. Conolly as their chief. Through his exertions and those of Sir William Burnett, we are justified in saying of HASLAR what Professor Marx so finely said of HANWELL; and we think the Professor's words, which we once proposed as a motto for Hanwell, may with full propriety be now inscribed on the walls of Haslar:—"HIER WIRD DURCH DIE THAT BEWIESEN WAS DER MENSCH UBER DEN MENSCHEN DURCH DAS MENSCHLICHE VERMAG."—*From No. XXXIII. of the British and Foreign Medical Review.*

---

### MEDICAL ETIQUETTE.

Dr. Carter, of Montreal, has related a case in which there was a decided breach of Medical Etiquette, and common prudence, too, provided the statement be correct. A young lady had had more than one attack of ileo-cœcal inflammation, swelling, and accumulation of fæces. These symptoms were subdued by bleeding, purging, &c. by which large quantities of scybala were evacuated. From certain symptoms that accompanied and followed these attacks, Dr. Carter believed that suppuration had, at one time, occurred. Of this we are not quite satisfied; but there was danger enough in these attacks to authorise the means employed. When the lady was convalescent, some of her friends suggested a consultation, which was agreed to by Dr. Carter. It appears that—

"Dr. Campbell, finding no abscess existing, entered into an argument *at the bed-side of the patient,* and maintained that it could never have existed." If this be a correct version of the affair, we must say that Dr. Campbell acted very indelicately and injudiciously. In the first place, the consultant who is called in to a case, should never contradict the statements, or even the opinions of the previous attendant before any of the patient's relatives, or the patient himself, even if he differs from his colleague. In the second place, he has no right to give a positive opinion of the past, but only of the present condition of a case. In the third place, he injures the profession, as well as his colleague—aye, and himself in the end, by remarks that tend to lead the patient or friends to suppose that any error has been committed either in practice or diagnosis.

We grieve to say, that these imprudent and illiberal, as well as unwise censures on each other, are too often indulged in by medical men, by which conduct, the public are naturally led to the conclusion that physic is a farce, as no two practitioners can agree as to the nature or treatment of a malady! This is the plague-spot on medicine. You cannot open a page in any of the periodicals without reading the most violent discussions, recriminations, and accusations bandied about between one practitioner and another! How can the public respect us, when we cannot respect each other?

---

VIVISECTION.—LETTER TO THE EARL OF CAERNARVON. From RICHARD JAMESON, ESQ.

It appears that Lord Caernarvon is President of the "SOCIETY FOR PREVENTING CRUELTY TO ANIMALS," and, as such, has been holding forth against the high crimes and misdemeanors of the medical profession for torturing animals to death in their physiological experiments! A certain Rev. J. Styles has gained a prize of £100. for the best Essay on this Pseudo-philanthropy, and has informed the public—"that every surgeon's apprentice thinks himself entitled to find his way into the arcana of Nature by scalping cats and rabbits *to see where their brains lie.*"

This is not merely a "*clerical* error," as the lawyers say, but a clerical lie. Admitting, for the sake of argument, that the medical profession should exhibit, among its numerous ranks, an instance or two of such senseless cruelty, is that any reason for using the sweeping assertion that "*every surgeon's apprentice, &c. &c.*" Does the ecclesiastical world never exhibit a black sheep?—If, because a Bishop was seen running down St. James's-street with his "*tights*" about his heels, we were to assert that every clergyman was a ———, what would this mendacious John Styles say to the accusation?

The only apology that can be offered for this Doctor of Divinity is, that he is a credulous ass. Thus, to astonish the Gaube-Mouches among his auditors, he tells them that oxen are driven many days without food, with "their hoofs worn off, and on *bleeding stumps!!*" Why Baron Munchausen was nothing to this D. D.! Can he be so silly too as to suppose that a grazier or a butcher would starve his cattle for days together, and thus so reduce their weight and condition as to suffer a considerable loss in the sale? This sapient philosopher tells us that the Hippopotamus, whose side would resist a musket bullet, has recourse to phlebotomy, when sick, by running against the point of a reed!! By way of contrast to the surgeons' apprentices, he informs us that predaceous animals destroy their victims with the least possible amount of pain, and that it is usually in the night time, when they are asleep, that they pounce on their prey, murdering them before they have time to reflect on their own danger! What tender-hearted creatures these tigers, hyenas, cats, and owls are! It is not because they can surprize their prey more easily in the dark than in the light, that they prowl about after sunset, but entirely to save the deer, goats, mice, and sparrows, the pain of being slaughtered! See how the cat domesticated among our amiable tabbies, fondles and soothes its victim the mouse, before it gives the final coup de grace!

But look at MAN himself. Leaving aside the wars, murders, and persecutions of all ages, see how many thousands of animals are annually slaughtered in the most cruel manner, by your nobility and gentry, while hunting the stag, the hare, &c. and that for mere sport, and without any regard to science or ultimate utility. The subject is extremely well handled by Mr. Jameson, and does great credit to his head and his heart. We strongly recommend its perusal.

---

### Instinct and Reason.

The distinction between these two attributes has afforded Dr. Strang, of Portsmouth, the subject for a lecture before the Philosophical and Literary Society of that place, in last October. Considering that the distinction in question has occupied the most able pens at various periods, Dr. Strang has thrown as much novelty and interest into the investigation as could possibly be expected. The broad general distinction between the two gifts of Nature or Nature's god, is visible and appreciable enough by all; but where the two attributes touch or become dovetailed, as it were, there is no small difficulty in saying where instinct ceases and reason takes the command. Lord Bacon considered that the faculties of man differed from those of animals, not only in *degree*, but in *kind;* whereas a modern Lord—Lord Brougham—looks on the difference as more in *degree*, resulting from the greater development of the human mind or its material organ. But man exhibits both instinct and reason. As an infant, the act of sucking is purely instinctive; and so the bee, when forming its hexagonal cells, acts without any reflexion, but by an impulse which it cannot resist. Reason is very different. It adapts means to ends, and *varies* them according to circumstances.

"Thus we never find two men work precisely alike—nor does the same man at all times evince the same degree of skill—but the bee made its cells as perfect 6000 years ago as it does at the present day, and all bees make them precisely in the same form."

We cannot entirely agree with our author respecting the operations of the beaver. That most sagacious animal constructs his domicile very differently, according to the nature of the locality; and when we find one party detached to the woods to cut timber—another employed in dragging it to the river or pond—a third, sharpening the stakes—a fourth driving them into the mud, and so on, with most admirable division of labour, we can hardly deny him a degree, and no small degree, of reason. We cannot coincide with Dr. Strang in his severe censures on those physiologists who have had recourse to vivisections for the purpose of ascertaining vital and animal functions. But that by the way. Dr. S. gives a great many instances of the reasoning powers in animals, especially the dog, some of which, if true, can leave no doubt that this boasted prerogative of man is shared in no small degree by the elephant, the dog, and other creatures called irrational.

There are many characteristic distinctions, however, between the human race and the brute creation. Instinct is stationary—reason is progressive—man knows that he must die—animals are free from that melancholy reflexion. Animals are tee-totallers—man is a drinker of intoxicating materials. Animals have neither hopes nor fears of a future state of existence—man has both. Animals rarely kill more game than they can eat—man kills twenty times as much as he can devour. Instinct never prompts to the worship of a Divine Being—reason prompts to the adoration of numerous gods under various forms. But there are various other distinctions, which we need not notice.

"We may conclude, then, that the functions which are simply vital and organic, and which are therefore necessary for even the lowest grade of sensitive existence, are possessed equally by all animals. In the distribution of the functions of animal life, in the degree of sensibility, and the power of voluntary motion, we observe a greater inequality in the various classes of animal existence. In neither of these, the organic and animal functions, does man obtain any superiority—he may even be inferior in these respects to many other animals. In strength of muscle or acuteness of sensation, he is in fact surpassed by many of them; but he is the *sole* possessor of those mental faculties that are the source of that vast superiority he enjoys over all other creatures here, and which must for ever separate him from them all."

Dr. Strang's lecture is very creditable to his attainments and good sense, and appears to have been extremely well adapted to the audience before which it was delivered.

---

### Fellowship of the Royal College of Surgeons.

The Committee of "The Medical Protection Assembly," so well known to all readers of the Medical Periodical Press, have addressed the following remonstrance to the Council of the College of Surgeons in reference to the election of Fellows vested in them.

*An Address from the Members of the Royal College of Surgeons of England to the Council of that College.*

Gentlemen,—In addressing you, at this vitally important juncture, as the governing body of the College of which we are members, we shall not offer to you either the language of remonstrance or complaint; but we feel it to be a duty which we alike owe to the public, to the Crown, to the members of the medical profession, and to ourselves, to remind you of the power which you have authority to exercise under the provisions of the Charter which her gracious Majesty granted to our College in the month of September, 1843.

In the *second* section of that royal decree you were empowered to elect, within three months from the date of the sign manual of the Sovereign, not less than two hundred and fifty, nor more than three hundred MEMBERS, who should, from thenceforth, become FELLOWS of our College. In the same section it is provided that *after* the expiration of three calendar months, and *within one year* from the date of the Charter "any *other persons* whom the Council shall *think fit and appoint*, shall be FELLOWS of the College."

We also request your attention to the *fourth* section of the said Charter, which we here present to your notice verbatim :—

"4. That it shall also be lawful for the Council of the said College, at any time or times after the expiration of the said three calendar months, and before the expiration of one year from the date hereof, by diploma or diplomas under the seal of the said College, and in such form as the said Council shall think fit, and without any fee, to appoint any other person or persons (being a member or members of the said College) to be a Fellow or Fellows of the said Royal College of Surgeons of England."

You have already selected, within the period prescribed by law, the three hundred members of our body, and created them FELLOWS of the College.

On the principles which have guided or regulated your choice in this selection we offer no comment; but we consider it to be our duty to remind you that *additional* creations of FELLOWS have not been announced by you, although *four* months have elapsed since the 14th of December last (when the first three months of your power to elect fellows terminated), and that there remain but five months of the entire term which the Charter has prescribed for the further exercise of your authority. Hence it has become our imperative duty to remind you of the power with which you are invested by the Crown.

The members of our College amount to upwards of ten thousand in number. For ought we know to the contrary, only three hundred of us have, up to this time, been created Fellows by you, under the provisions of the new Charter. This is rather an alarming result of a seven months' exercise of your creative power.

The *Fellows,* be it remembered, are to be the future *electors* of the Council.

The privilege of *election* is a right which we, the members, have endeavoured to obtain from the legislature during nearly twenty years. Instead of conferring it upon *us,* directly, the Crown has invested *you* with full authority to enfranchise us. The Crown rightly judged, perhaps, that *you*, who granted us our diplomas, must be better acquainted with our characters and capabilities than could be any body of unprofessional persons with whom we had never been intimately associated.

The fact, in truth, cannot be concealed, that *you* have authority to enfranchise *every member of the College who resides within the limits of this kingdom.* You might even bestow the fellowship on members who live beyond those limits. For the proofs of these allegations we refer to the sections already quoted, in which it is distinctly stated that it shall be lawful for the Council, within twelve months from the date of the Charter (14th September, 1843), to appoint, without fee, "any other person or persons, being a member of the said College, to be a Fellow or Fellows of the said Royal College of Surgeons of England."

These words admit not of two interpretations. They possess an obvious, a perfectly unequivocal meaning. The authority to create *thousands* of Fellows from among the existing members of the College is by law vested *with you*, the Council.

Thus, the power to enfranchise us, to bestow upon us the right of electing the future Councillors of the College, *now exists with you.* It will *expire* on the 14th of September next. We think, then, that we are justified in demanding at your hands that it shall be exercised promptly, comprehensively, liberally, justly; and we venture to express an anxious hope that the employment of it, by you, will not be tainted either by prejudices or dishonest partialities.

We refer to this new *Charter*, which has invested you with so much authority, as to a highly important sign in the present crises of our affairs. We respectfully remind you that as *you* are now empowered by law to bestow upon us the right of enfranchisement, so upon *you* will fall the whole weight, responsibility and disgrace, which would attend an unconstitutional, illiberal, unjust exercise of the unusual functions with which you have been invested by the Crown. We have the honour to be, Gentlemen, your's, &c. &c.

This has been followed by a "Statement," on the part of the Council of the College, which we also subjoin.

*Statement relating to the Charter lately granted by Her Majesty to the Royal College of Surgeons of England.*

The Council of the Royal College of Surgeons of England feel that the time is arrived when it is proper for them to offer some observations, in explanation of the principal changes which the Charter, lately granted by her Majesty, has occasioned in the constitution of the College, and on the ultimate effect which these changes may be expected to produce in the condition of the Surgical profession. They avail themselves of the opportunity thus afforded to state the principles on which they have hitherto acted, and those on which they propose to act hereafter in the exercise of the new duties which this Charter has imposed upon them.

The Bye-laws which may hereafter be made for the government of the College will not be valid until approved of by the Crown.

The Members of the Council will be elected, not for life, but for a limited term of years.

When vacancies occur in the Council they will be supplied, not by the Council, but by the new body of Fellows, who will elect the new Members from among themselves.

Fellows of the College, who are not Members of the Council, will be equally eligible to the Court of Examiners with those who are; and future Examiners will hold their office only during the pleasure of the Council.

One object of Her Majesty's Advisers, in establishing the Class of Fellows, was to create a sufficient Constituency for the election of the Council. The same end might have been attained by simpler means, such as giving the franchise to members of a certain standing in the College; but another and expressly avowed purpose was to promote a spirit of emulation among Surgeons, to afford additional inducements to exertion in the cultivation of science, and thus to increase the utility and elevate the character of the Surgical profession. After the expiration of one year from the date of the Charter, no one will be admitted into the rank of Fellows, until he has undergone a strict and lengthened examination, not only in practical surgery, but also in the collateral sciences. They who aspire to become Fellows, without having been previously Members of the College, will be required to have gone through an extended course of professional study in Hospitals and Schools, and to be at least twenty-five years of age. But the Fellowship will not be limited to Candidates of this description; and they who, not having had the same advantages of education, have been admitted as members at twenty-one years of age, may, after having been engaged in Practice for a certain number of years, represent to the Council that they have continued to study their profession as a science, and claim on these grounds to be examined for the Fellowship. Thus any individual, however limited his means of improvement may have been in early life, may raise himself by his own industry and talents to the same rank in the College with those who were in the first instance more fortunately situated. No one who desires to attain the Fellowship can complain that it is not within his reach, or that he is prevented from becoming an Elector, or a Member of the Council, or of the Court of Examiners.

Candidates for the Fellowship at twenty-five years of age, will have had the opportunity of obtaining a liberal general education, previously to entering on the studies peculiar to their profession; and it is reasonable to expect that the example of such well-educated persons will influence those, whose preliminary education has been imperfect, to supply the deficiency by devoting to the acquirement of various knowledge and the general cultivation of the mind a portion of the leisure which falls to the lot of every young practitioner.

The course to be pursued in the future admission of Fellows is sufficiently obvious; but the new Charter imposed upon the Council another task of much greater difficulty, that of selecting, from among the many thousand Members of the College, a limited number of individuals to be nominated as Fellows in the first instance, so as to form an immediate constituency for the future election of Members of the Council.

The following provisions of the Charter, on this subject, give to the Council the absolute power of nomination, without conferring on any description of Members the right to be so nominated.

" The Council of the said College, with all convenient speed after the date of these Our Letters Patent, and before the expiration of three calendar months from the date hereof, and in such manner as the said Council shall deem best, shall elect to be Fellows of the said College any such number of persons, being Members of the said College, and not being in the whole less than 250 nor more than 300, as the said Council shall think proper."

" It shall also be lawful for the Council of the said College, at any time or times, after the expiration of the said three calendar months, and before the expiration of one year from the date hereof, by diploma or diplomas under the seal of the said College, and in such form as the said Council shall think fit, and without any fee, to appoint any other person or persons, being a Member or Members of the said College, to be a Fellow or Fellows of the said Royal College of Surgeons of England."

When these passages of the Charter are considered in reference to each other, and in combination with the circumstances that the nomination of Fellows by the Council is only a temporary expedient, designed to provide, in the first instance, that Constituency which will be supplied hereafter by Fellows admitted on examination, it will be obvious that the framers of the Charter did not intend that the Fellows to be thus nominated should greatly exceed the number of three hundred.

The Council entered on the duty assigned to them by these provisions of the Charter with a full sense of its invidious nature. They were aware that of those not included in the list of Fellows, a considerable number would feel and express dissatisfaction. But they have done what was required of them to the best of their ability, and have made the selection altogether on public grounds, without favour or prejudice, and uninfluenced by private motives. The following statement will sufficiently explain the principles on which they have acted.

The great majority of the Members of this College are less engaged in the practice of Surgery than in that of Medicine, Midwifery, and Pharmacy, and many of them have arrived at well-deserved eminence in these latter departments of the Medical profession. But the Council, keeping in view the objects for which the College was especially established, have felt it their duty, in the nomination of Fellows, to regard chiefly the qualifications of Members as practitioners in Surgery, or as improvers of those sciences which tend to its advancement.

1. In accordance with this principle, they placed in the list of Fellows the Surgeons of all the Hospitals in England and Wales which are recognised by them as schools of Surgery; and they did so under the conviction that the Surgeons of large Hospitals have the best opportunity of experience in Surgery, and that they are the persons principally consulted in private practice, and referred to by other practitioners, in surgical cases.

2. But they were aware that in several parts of the kingdom there are Members of the College having considerable reputation as Surgeons, and called into consultation in surgical cases by the practitioners in their neighbourhood, although they have no connexion with Hospitals; and the Council thought it right to place the most eminent of such persons on the list of Fellows. In executing this part of their duties great circumspection was required, lest improper names should be inserted and proper ones omitted. In this respect the list is incomplete, there being individuals of this class whose claims are still under consideration.

3. Not being well acquainted with the qualifications of Military and Naval Surgeons, and being at the same time desirous of doing justice to them, the Council applied for assistance to the heads of their respective departments; and many of the names included in the schedule of Fellows are the result of this application.

4. There are in London several practitioners in Surgery, who, though not connected with Hospitals, were considered eligible to the Council under the former Charter and according to former usages, and the Council therefore thought that they ought to be admitted to the Fellowship. Many of these gentlemen are well known and much esteemed by the profession; and the question was, not whether they should be elevated to a new position, but whether they should be displaced from one which they had previously occupied.

5. Some individuals have been placed on the list of Fellows from having distinguished themselves in cultivating the kindred sciences of Anatomy, Physiology, and Natural History. The Council cannot but regard such persons as ornaments of the College, and it will be gratifying to them to find others of the same class who may be added to the list.

6. Other names have been inserted for special reasons, being principally those of Teachers who had been recognised by former acts of the Council, or of persons holding important public offices. Among the latter are four Senators of the University of London.

The Council are empowered to nominate an additional number of Fellows before the expiration of the first year from the date of the Charter. This will enable them to supply the deficiencies of the former list, in anticipation of which it is evident that this clause was introduced. In the future nomination of Fellows, the Council see no reason why they should depart from the general principles on which they have hitherto acted; though they will make it their object to omit the name of no individual who is held in esteem by the other Members of the College for his surgical experience and scientific attainments. For those who are not yet so distinguished, there is an honourable method of obtaining the Fellowship by examination.

In conclusion, the Council take the liberty of observing, that no alteration in the Charter, nor any legislative enactment, can materially change the condition of those who have been for some time established in practice. In the Medical profession each individual makes his own place in society; intellect, knowledge and integrity being equally appreciated and respected in every grade and station. If the changes introduced by the present Charter are to have the effect of elevating the character of the Surgical profession generally, it will be in the next rather than in the present generation; and if the elder practitioners are interested in these changes, it is less on their own account, than on that of their sons and successors.

By order of the Council,
EDMUND BELFOUR,
Secretary.

Lincoln's Inn Fields, May 25, 1844.

Setting aside the justice or injustice, propriety or impropriety of the Charter itself, and discussing merely the question raised in the Address of the Medical Protection Assembly, there cannot be a doubt that the Council of the College has the best of the argument. It must be clear to every one, from the limitation of the numbers of the first batch of Fellows, that the Crown did not design the body to be numerous. If any inference is to be deduced from the general spirit of the Charter, it points too the same way. Its aim is obviously to create a small and select body, large enough to comprise the distinguished members of the College, but not so large as to be held cheap, from the facilities of belonging to it. This we say, special pleading apart, is clearly the intention of the framers of the Charter, and the Council unanswerably appeal to it.

We have already stated that we are not now discussing the merits or demerits of the Charter itself. It might or it might not have been a better one. But it is " a fact," and as such we take it. Nor are we discussing the principles which have guided the Council in the selection of the " Fellows." The " Protection Assembly" waive an examination of those principles, and so do we. We content ourselves with one observation—that *if* there are to be Fellows at all, the Fellowship ought not to be made contemptible by being swamped with numbers—and that the power vested in the Council, being in its nature highly responsible and equally invidious, ought to be exercised with strict impartiality, untainted by the suspicion of sinister influence, jobbing, and class-feeling.

---

### Statement of the Society of Apothecaries, &c.

This is in the form of a Letter to Sir James Graham, from the Company, setting forth what they have done during the last thirty years, in the way of improving the education of the general practitioner. They combat the objection to an examination being entrusted to a Court of his own class, instead of one of a higher grade—as that of the College of Surgeons, or of Physicians, by shewing the Curriculum which they now enforce, and the qualifications they now require in the candidate, as compared with the regulations which they first enacted in 1815. The contrast is indeed most striking, and shews the rapid advance that medical education has made in a quarter of a century. The Curriculum of the Apothecaries' Company at the present moment, would have been an almost impassable barrier to any medical aspirant in the first years of the nineteenth century.

Gradual as has been the extension of the Curriculum, since 1815, the Court has "plucked" no less than 1,531 of the candidates for admission into the practice of physick! This was about one in eight of the applicants. There can be no doubt, indeed, that the Apothecaries' Company have discharged their duties with great propriety and assiduity; but still the opinion is gaining ground daily, that there should be *one* governing body, not only for regulating the Curriculum of study, but for examining the candidates for the diploma in the three branches of the profession. The examinators would, of course, be selected from the three existing bodies, but the Apothecaries seem to fear that theirs would not be the lion's share of the spoil.

"An apprehension appears to exist on the part of many, that it will be proposed to Parliament to exclude the general practitioner from all share in the examination of his own class, or to place him in so small a minority at the examining board, as to deprive him of all real control and influence in a matter in which the interests of his branch of the profession are so deeply involved."

But supposing that the Court of Examiners consisted of nine persons—three from each of the existing Corporations—we do not see that the Apothecaries' Company would be in a minority. It is far more likely that the delegates from one or other of the two higher bodies would side with the Apothecaries, on any disputed point, than that they would coalesce against the representatives of the general practitioners. In any clash between the physicians and surgeons, the apothecaries would have the casting vote; and, as is sometimes the case in politics, "*the tail would turn the scale.*" But it is needless to speculate, since the Government measure is, at the time we write, little known. It requires no gift of prophecy, however, to predict that it will be far from giving satisfaction to even a majority of the heterogeneous mass of medical society, as it now exists.

---

### CASE OF VERY LARGE STONE IN THE BLADDER EXTRACTED THROUGH THE PERINEUM.

The following case which has been transmitted to us, is one of much surgical interest. We have abbreviated the less material details.

John M'Gregor, Corporal in the Mounted Coast Guard, aged 49, entered Melville Hospital, Chatham, with symptoms of stone in the bladder, which he had laboured under, more or less, from June 1813. On sounding him on the 18th June, 1843, Dr. Rae, Deputy Inspector, found a large stone, round the apex only of which the instrument could pass, and examined by the rectum, the finger could neither raise nor pass beyond the stone. The patient himself declared that he frequently felt the stone move in the bladder, and that he could frequently retain his urine for two hours. Under these circumstances, Dr. Rae determined to perform the lateral operation. This we give in his own words.

The staff being introduced, and the patient secured in the usual way, I began my incision nearly two inches above the anus, and a quarter of an inch to the left of the raphe, at once an inch deep, rendering it more superficial as I passed the anus; the cellular membrane was then farther divided, and the transversus perinei muscle; my finger was then without difficulty pushed in to the deep-seated fascia, the groove of the staff felt for and cut into; a probe-pointed knife was now substituted for the former one, fairly inserted into the groove, carried on to the bladder, and next moment my finger was on the stone. Had this been of the usual magnitude, the patient would have been replaced in bed in a quarter of an hour with the loss of not six ounces of blood; but now all our difficulties began, and I was convinced that the patient could not have felt the stone moving for years, nor could he have retained his urine for two hours, indeed he has since informed me that he could not keep it more than a quarter of an hour during the day, or half an hour at night, and that he concealed his complaint as much as possible. The stone was now found to be of great size, over which the thickened coats of the bladder were permanently contracted. It seemed to be of a conical shape, the apex only being free, and to pass the forceps over it, and expand them for the purpose of extraction, was perfectly impracticable, the blades actually bent, and had I used more force, in the opinion of all present, I must have lacerated the bladder; the small end of a scoop could not pass without danger of perforation. The lower part only could be grasped, and the forceps almost invariably slipped, bringing away debris and numerous fragments each time. After many and repeated trials to extract, I handed the forceps to my able assistant, Dr. Charlton, but with no better success. The patient had now been an hour and twenty minutes on the table, and although he bore his suffering with heroic firmness, worthy of a M'Gregor, still he was getting exhausted, and it became absolutely necessary to desist. The bladder was washed out with warm water, and an elastic tube with lint placed in the wound, and he was carried pulseless to bed; warm brandy punch was administered, and warmth applied to the feet: an anodyne was afterwards given, and in a short time he rallied."

He recovered from the effects of the operation marvellously. The remainder of the case is instructive.

The wound was studiously kept open for farther proceedings, the stone was occasionally felt, and as had been predicted by my friend Mr. Fergusson of King's College, to whom I had related the case, became looser and less firmly embraced by the bladder. During this interval the Director General, Sir Wm. Burnett, had ordered at my request, a pair of forceps to be made very strong, and with separate blades like those for midwifery,* with which I expected either to crush or extract. Having been in town, however, I called upon Mr. Guthrie, who kindly directed Mr. Simpson of the Strand, to make for me a crushing instrument, which he thought would suffice to break the calculus. Mr. Fergusson, same day, lent me one still more powerful, but on trial, neither of them had sufficient space between their prongs to grasp the stone, whose longest diameter was between the fundus and prostate. While waiting for my new forceps, I received a note from Mr. Fergusson, of 27th Jan. saying that he was called to see a lady at Chatham, would be down the following day, and, in case I had not succeeded in applying the crusher, would bring two fresh instruments with him. He came accordingly, Jan. 28th, bringing two pairs of forceps lined in their points, and one with a peculiar hinge, with which he had extracted several large stones. On minutely examining the situation and size of the calculus, both by the wound and rectum, Mr. F. was of opinion that it might then be extracted, though probably with some difficulty. Whereupon I waived all ceremony or selfish consideration, felt for the safty and welfare of my patient, was glad to avail myself of Mr. F.'s experience, skill, and dexterity in such cases, and requested he would proceed to extract. He kindly complied, little preparation was necessary, the incisions were already made, he introduced a pair of his own forceps and was not long in extracting, though he met with much resistance, and the forceps slipped twice. Several broken pieces were extracted by the scoop, the bladder was washed out with tepid water and the patient placed in bed, complaining of much pain in the wound and in his back, and subsequently had a rigor. Some wine was given, and a morph. draught at bed-time. He passed a good night, and in the morning was free from pain or fever. Pulse 96, soft and small. Bladder washed out with tepid water injected by the penis. To have wine and arrow-root.

---

* Mr. Simpson has now completed this instrument, and has ingeniously added to it a drill or perforator. It has been highly approved of by several eminent surgeons in London, and promises to be of great utility in cases of large calculi.

The stone itself, including the fragments, weighed nearly eight ounces, and, allowing half an ounce for the loss (though my assistant and others say there was more than a full ounce), the stone must originally have been nearly nine ounces in weight. It was smooth externally, and composed of circular laminæ, seemingly of triple phosphate formation, but hard and firm in its interior part. To its smooth surface, and the firm manner in which it was held by the bladder, may be attributed the comparative ease with which the patient rode on horseback for such a number of years, and which a loose body of such magnitude must have rendered impossible. This case has caused me much anxiety, yet I do not regret that the stone was not extracted on the first occasion, considering the difficulties I had to contend with, and had I persisted longer, or used more force to accomplish my object, the patient must have died from exhaustion and great constitutional injury. By delay, allowing him to recover himself, and keeping the wound open for future proceedings, all has done well, and although I am indebted to the opportune arrival of Mr. Fergusson, yet I feel confident that I would have been able to extract or crush with the forceps which Mr. Simpson has now completed for me.

The issue was most satisfactory. On the 5th March, it is reported that for three days no urine had passed by the wound, which was nearly cicatrised. On the 19th of April he was discharged perfectly cured.

---

### MEDICAL REFORM BILL.

The prominent features—we might say the "form and pressure" of this long-expected Millennium-medicine have oozed out, so that its actual advent will not cause us to "burst with ignorance." We doubt whether the enactment of the new law will give general satisfaction, though we believe that its operation will be beneficial to the public. The main, we might almost say the whole tendency of the proposed Bill, is to raise and render uniform the education of the general practitioner—for it scarcely touches or notices the various Colleges and Corporate Bodies which now grant diplomas to practise medicine and surgery throughout the kingdoms.

Mr. Carmichael, who was in epistolary correspondence with Sir Benjamin Brodie during the incubation of the forthcoming measure, delivered an address to the Irish Medical Association, from which the following extracts are made.

"Although this Bill does not proceed to the full extent in reforming the profession that we deemed necessary at our former meetings, yet it fulfils our expectations to a very considerable extent, and will, no doubt, be found to confer important benefits upon society, while it will increase, in no slight degree, the utility and respectability of the medical profession in all its departments. It certainly does not, as we desired, unite physic and surgery into one faculty, by combining the Colleges of Physicians and Surgeons; but it enacts that all general practitioners shall, in future, undergo sufficient examinations in both physic and surgery for all practical purposes; and, as we may fairly estimate that nine-tenths of all medical and surgical cases in the united kingdom are *now* in the hands of general practitioners, *the union of physic and surgery*—one of the grand objects of medical reformers—is therefore all but established by this Bill.

"Neither does it in a decided way *separate, as we desired, the practice of pharmacy from the practice of medicine;* but the respectable portion of the general practitioners of England—and there are amongst them men of the highest medical attainments—no longer vend medicines; and I am informed, from the best authority, that the practice of keeping open shop for the sale of medicines is gradually getting into disrepute and disuse amongst them, and will, it is expected, soon cease altogether. Besides, the druggists of England have lately obtained a charter empowering them to compound medicine. They will not be qualified or licensed to practise medicine, and therefore this distinct charter for pharmacy alone will tend, in no slight degree, to *separate the practice of pharmacy from the practice of medicine.* The cross-counter practice, however, as it is called, of English druggists, must be interdicted by some stringent regulations.

" Sir James Graham's Bill will not deprive, as we wished, the nineteen or twenty medical colleges or corporations of the licensing power, and substitute in their place three others in London, Dublin, and Edinburgh ; but it will put a complete and efficient check upon venality and malpractices, by the establishment of a Central Council, with powers to regulate the examinations, and to dictate the quantum of qualification to be expected from candidates who seek to enter into the profession. This Council will therefore have the power of raising and equalizing the standard of medical education through every portion of the empire ; so that the licensed and registered practitioners of England, Ireland, and Scotland, will be on a level, and qualified to fill any medical situation in the three countries ; and thus another of the great objects of medical reformers will be obtained by this Bill—*Equality of qualification and privileges throughout the empire.*"

" This Bill will also deprive the Apothecaries' Company of London of the power of granting licenses to practise medicine. This power was permitted in 1815 by an act of the Legislature, which a correspondent of the *Times*' newspaper describes to have been 'smuggled through parliament to aggrandize a trading company, and *annihilated the respectability of the medical profession, and made it a mean calling ;* for, unfortunately for the public, it has been the instrument of transferring the medical care of the sick—in every rank and station—almost entirely from the regularly-educated prescribing surgeon and physician, to the trading practitioner, whose system of drugging and drenching the dupes who employ him is a heavier burden upon the industrious and struggling classes than the poor-rate and income-tax both together.' We must agree with this writer; but, after the passing of Sir James's Bill, the Apothecaries' Company of London will be relieved from duties for which it was unfitted, and which it should never have undertaken, and will have more leisure to cultivate pharmacy, with its accessory sciences—chemistry, botany, and natural history."—*Dublin Medical Press.*

On learning the foregoing particulars, Mr. Carmichael put himself in epistolary communication with Sir B. Brodie, and wrote him a long letter, suggesting many alterations, and, as he thought, improvements. The main modification was that of separating pharmacy from the practice of medicine and surgery—or, if that could not be done, to enable the practitioner to charge for his visits *only,* and include the medicines gratis. This plan, he thinks, would obviate all objections, and do entirely away with the vile practice of throwing in too much physic, as the sole chargeable article according to the existing law. We have often shewn that this attempt to include the physic in the fee would be abortive, inexpedient, or even impracticable. The present plan of making the medicines *only* chargeable in the bill, is, no doubt, an evil, leading to overdosing. But the other extreme of charging only for the visits, would give equal discontent. It is to be remembered that nine-tenths of the sick, whether rich or poor, are grumblers and ingrates. If we charge for the physic alone, then they accuse us of pouring down their throats three times as much as is necessary—if we charge for visits, *including medicine,* then we send them too little, or what is worse, we give them nothing but the coarsest and cheapest drugs, scarcely fit for dogs or horses, while we pay them far more visits than are necessary! The only effectual remedy, as we have often stated, is the dissociation of physic from pharmacy. Suppose the law enabled the practitioner to charge five shillings for his visit, including medicine: would it not be infinitely better for him to receive 3s. 6d. for his visit, letting the 1s. 6d, go to the chemist who prepares the medicine?

The new law will raise the education of the general practitioner, as nearly as possible, to the level of the pure physician and the pure surgeon, and leave him still in the degrading position of dispensing as well as prescribing his own medicines. It will be beneficial to the public, however, by equalizing as well as elevating the standard of professional attainments.

---

### THE LATE WILLIAM ALLEN.

The Vice President, in his address, gave a slight sketch of the career of Mr. William Allen, lately deceased. Mr. W. Allen, born Aug. 29, 1770, was the son of Mr. Job Allen, a member of the Society of Friends, and a silk manufacturer in Spitalfields. Mr. W. Allen, having in early life evinced a taste for chemical pursuits, was placed under the care of Mr. J. Gurney Bevan, under whose care he first acquired a practical knowledge of chemistry, and whom he afterwards succeeded in the concern. Here he was accustomed to rise at four or five o'clock in the morning, and sedulously apply himself to study, the usual hours of business being fully occupied. About the year 1804, he became connected as a public lecturer with the Medical School of Guy's Hospital, his coadjutors in the lectures being successively the late Dr. Babington, then the late Dr. Marcet, and afterwards Dr. Bostock. About this time also, he accepted the chair of Experimental Philosophy at the Royal Institution, which he held for several years. His talents as a philosopher brought him into habits of intimacy with the most distinguished scientific men of the day, Sir H. Davy, John Dalton, &c. His most intimate associate and friend was Wm.. Haseldine Pepys, with whom he was for several years engaged in chemical investigations. The most prominent of these were communicated to the Royal Society, and printed in the Philosophical Transactions of 1807, 1808 and 1809. Of this Society he was elected a Fellow in 1807. After a life spent in the active pursuits of science, he died in a good old age, at the age of 74, on the 30th of last January.—*Pharmaceutical Journal,* Feb. 1, 1844.

